# An induced pluripotent stem cell line (TRNDi006-A) from a MPS IIIB patient carrying homozygous mutation of p.Glu153Lys in the *NAGLU* gene

**DOI:** 10.1016/j.scr.2019.101427

**Published:** 2019-03-23

**Authors:** Wei Huang, Miao Xu, Rong Li, Amanda Baskfield, Jennifer Kouznetsova, Jeanette Beers, Jizhong Zou, Chengyu Liu, Wei Zheng

**Affiliations:** a National Center for Advancing Translational Sciences, National Institutes of Health, Bethesda, MD, USA; b iPSC core, National Heart, Lung and Blood Institute, National Institutes of Health, Bethesda, MD, USA; c Transgenic Core, National Heart, Lung and Blood Institute, National Institutes of Health, Bethesda, MD, USA

## Abstract

Mucopolysaccharidosis type III B (MPS IIIB) is a lysosomal storage disorder caused by mutations in the *NAGLU* gene encoding *N*-acetylglucosaminidase. Here, we report the generation of a human induced pluripotent stem cell (iPSC) line from dermal fibroblasts of a MPS IIIB patient. The iPSC line has homozygous mutations of G > A transversion at nucleotide 457 of the *NAGLU* gene (457G > A), resulting in the substitution of lysine for glutamic acid at codon 153 (Glu153Lys). This iPSC line allows for the study of disease phenotypes and pathophysiology as well as disease modeling in human cells.

**Table:** Resource table

Unique stem cell line identifier	TRNDi006-A
Alternative name(s) of stem cell line	HT527A
Institution	National Institutes of Health National Center for Advancing Translational Sciences Bethesda, Maryland, USA
Contact information of distributor	Dr. Wei Zheng Wei.Zheng@nih.gov
Type of cell line	iPSC
Origin	Human
Additional origin info	Age: 1-year-old Sex: Female Ethnicity: Caucasian
Cell Source	Dermal fibroblasts
Clonality	Clonal
Method of reprogramming	Integration-free Sendai viral vectors
Genetic Modification	NO
Type of Modification	N/A
Associated disease	Mucopolysaccharidosis type III B (MPS IIIB)
Gene/locus	*NAGLU* ^Glu153Lys^
Method of modification	N/A
Name of transgene or resistance	N/A
Inducible/constitutive system	N/A
Date archived/stock date	2018
Cell line repository/bank	N/A
Ethical approval	NIGMS Informed Consent Form was obtained from patient at time of sample submission. Confidentiality Certificate: CC-GM-15-004

## Resource utility

This TRNDi006-A iPSC line is a valuable resource for elucidating the disease phenotype and pathophysiology of MPS IIIB. It can be differentiated into various mature cell types for use as cell-based disease models of MPS IIIB for compound screening and drug development.

## Resource details

MPS IIIB, also known as Sanfilippo syndrome type B, is an inherited lysosomal storage disease caused by mutations in the *NAGLU* gene, which encodes *N*-acetylglucosaminidase, which normally degrades heparin sulfate (Genger et al., 2018). Heparin sulfate proteoglycans can bind to many ligands to modulate various cellular activities and maintain tissue architecture and physiology. Deficiency of *N*-acet-ylglucosaminidase’s function causes lysosomal accumulation of heparin sulfate resulting in neurological dysfunction in MPS IIIB patients (Andrade et al., 2015), though the exact mechanism of MPS IIIB disease is unclear. Children with MPS IIIB have severe neurological and behavioral defects, leading to death in the second or third decade of life. Currently, there are no effective treatments for MPS IIIB. The clinical treatments for this disease are symptomatic and palliative that do not improve patient prognosis.

**Fig. 1.**
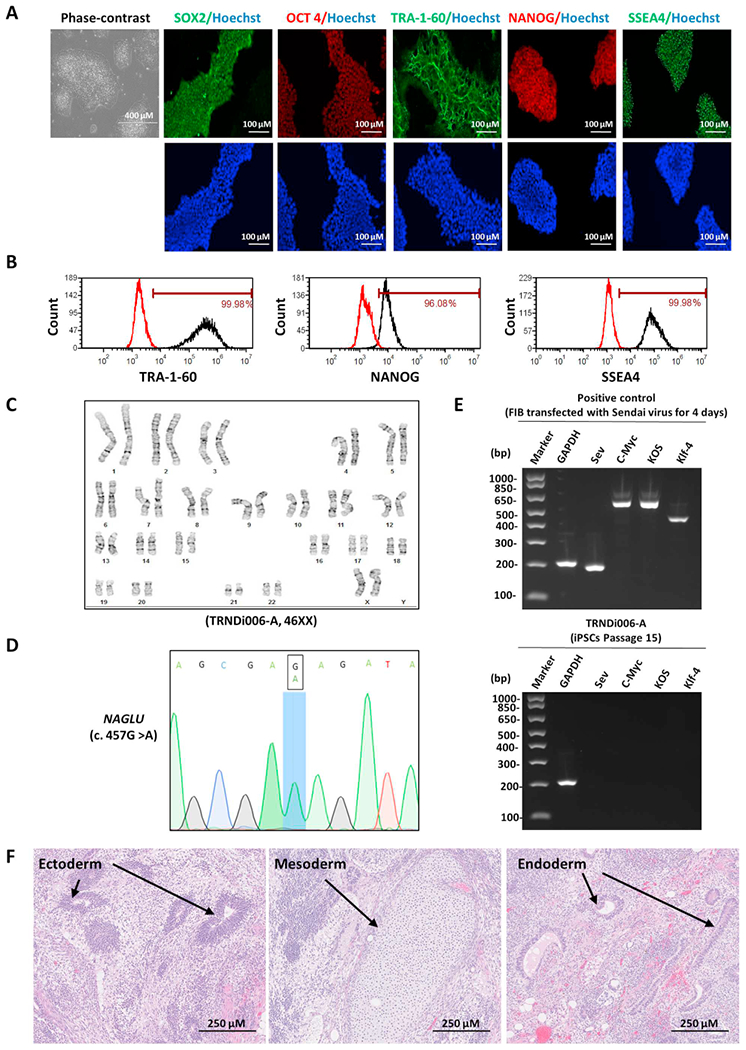
Characterization of TRNDi006-A iPSC line A) Left panel shows phase contrast imaging of TRNDi006-A colonies grown on Matrigel at passage 10; Right panels show immunofluorescent staining of TRNDi006-A iPSCs, demonstrating expression of SOX2, OCT4, TRA-1-60, NANOG and SSEA4. Hoechst (blue) was used to label the nucleus. B) Pluripotency protein markers TRA-1-60, NANOG and SSEA4 were assessed by flow cytometry analysis. C) Normal karyotype was confirmed through G-banding karyotype analysis (46, XX). D) A homozygous gene mutation of p.Glu153Lys (c.457 G > A) in the NAGLU gene was confirmed by Sanger sequencing. E) RT-PCR verification of the clearance of Sendai virus from the reprogrammed cells. Fibroblasts transfected with Sendai virus were used as positive control. F) Histological characterization of teratoma formation from TRNDi006-A, showing formation of three germ layers (Ectoderm, Mesoderm, and Endoderm).

In this study, an iPSC line was established from skin fibroblasts of a 1-year-old female patient carrying a homozygous gene mutation of p.Glu153Lys (c.457 G > A) in the *NAGLU* gene by using a non-integrating Sendai viral vector kit (A16517, ThermoFisher) containing OCT¾, KLF4, SOX2 and C-MYC pluripotency transcription factors (Beers et al., 2012; Beers et al., 2015). Mutations of the *NAGLU* gene in the newly generated iPSC line, designated as TRNDi006-A, were confirmed by Sanger sequencing of the PCR product harboring the single nucleotide variation (SNV) (Fig. 1D). The patient-derived iPS cells exhibited classical embryonic stem cell morphology (Fig. 1A) and a normal karyotype (46, XX) that was confirmed by G-banded karyotyping at passage 15 (Fig 1C). The cells expressed the major plur- ipotent protein markers of NANOG, SOX2, OCT4, SSEA4 and TRA-1–60 (Fig. 1 A, B) as evidenced by both immunofluorescence staining and flow cytometry analysis. Sendai virus vector (SeV) clearance was detected with reverse transcription polymerase chain reaction (RT-PCR) using SeV-specific primers with no virus present by passage 15 (Fig. 1E). Mycoplasma testing was performed for the TRNDi006-A cell line and a negative result was obtained (Supplementary Fig. S1). The iPSC line was authenticated using STR DNA profiling analysis which demonstrated matching genotypes at all 18 loci examined (information available from the authors). Furthermore, a teratoma formation experiment demonstrated pluripotency of this iPS cell line, as it exhibited its ability to differentiate into tissues of all three germ layers (ectoderm, neural tube; mesoderm, cartilage; endoderm, gut) *in vivo* (Fig. 1F). (See Table 1.)

## Materials and methods

### Cell culture

Patient skin fibroblasts, obtained from Coriell Institute (GM01426), were cultured in DMEM supplemented with 10% fetal bovine serum, 100 units/ml penicillin and 100μg/ml streptomycin in a humidified incubator with 5% CO_2_ at 37 °C. The iPSCs were cultured in StemFlex medium (ThermoFisher) on matrigel (Corning, 354277)-coated plates at 37 °C in humidified incubator with 5% CO_2_ and 5% O_2_. Cells were passaged with the dissociation agent of 0.5 mM ethylenediaminetetraacetic acid (EDTA) at 80% confluency.

### Reprogramming of human skin fibroblasts

Non-integrating Sendai virus was used to reprogram patient-derived fibroblasts into iPS cells. Methods were described previously (Beers et al., 2012; Beers et al., 2015).

### Genome analysis

Genomic analysis of *NAGLU* variants was performed by Applied StemCell (Milpitas, California). Genomic DNA was extracted from TRNDi006-A using QuickExtract™ DNA Extraction Solution (Lucigen) and PCR amplifications using MyTaq™ Red Mix (Bioline) were carried out using a previously defined protocol (Li et al., 2018). Sanger sequencing analysis was used for genotyping of the homozygous mutation for a p. Glu153Lys variant (c.457 G > A) of the *NAGLU* gene. The specific primers used in these protocols are listed in Table 2.

### Immunocytochemistry

The iPSCs were fixed with 4% paraformaldehyde, and permeabi- lized with 0.3% Triton X-100. Cells were treated overnight at 4 °C with SOX2, OCT4, NANOG, SSEA4 and TRA-1-60 primary antibodies (Table 2). After washing, cells were incubated with secondary antibodies conjugated with Alexa Fluor 488 or Alex Fluor 594. Cells were stained with Hoechst 33342 and imaged with an INCell Analyzer 2200 imaging system (GE Healthcare) using 20× objectives and Texas Red, FITC, and DAPI filter sets.

### Flow cytometry analysis

Cells were dissociated using TrypLE (ThermoFisher), fixed with 4% paraformaldehyde. Prior to fluorescence-activated cell sorting, cells were permeabilized with 0.2% Tween-20 in Dulbecco’s Phosphate Buffered Saline (DPBS)D and stained with fluorophore-conjugated antibodies for 1 h at 4°C on a shaker. Relative fluorophore-conjugated animal nonimmune immunoglobulin were used as the negative control (Antibodies and nonimmune immunoglobulin used are listed in Table 2). Cells were then analyzed on Accuri™ C6 Flow Cytometry system (BD Biosciences).

### G-banded karyotyping

The G-banding karyotype analysis was conducted at WiCell Research Institute (Madison, WI, USA). A total of 20 randomly selected metaphases were analyzed by G-banding for each cell line.

### Short tandem repeat (STR) analysis

Samples for cell line authentication were analyzed by the Johns Hopkins University Genetic Resources core facility using a PowerPlex 18D Kit (Promega) and the PCR product was electrophoresed on an ABI Prism® 3730×1 Genetic Analyzer. Data were analyzed using GeneMapper® v 4.0 software (Applied Biosystems).

### Mycoplasma detection

Mycoplasma status was assessed using the Lonza MycoAlert kit following the manufacturer protocol. A ratio of B/A < 0.9 indicates a mycoplasma negative sample.

### Sendai virus detection

For positive controls, human fibroblasts (Coriell, GM05659) were transfected with Sendai virus for 4 days, and total RNAs were extracted using RNeasy Plus Mini Kit (Qiagen). The cDNA was reverse transcribed using 1 μg of RNA with the Superscript™ III First-Strand Synthesis SuperMix kit and amplification was performed using Platinum II Hot-Start PCR Master Mix (ThermoFisher) with the following program: 94 °C, 2 mins; 30 cycles of [94 °C, 15 s, 60 °C, 15 s and 68 °C, 15 s] on the Mastercycler pro S (Eppendorf) and primers are listed in Table 2. Products were loaded to the E-Gel® 1.2% with SYBR Safe™ gel, and imaged using the G: Box Chemi-XX6 gel doc system (Syngene).

### Teratoma formation assay

The iPSCs were cultured in 6 well plates prior to dissociation with 0.5 mM EDTA in DPBS. Dissociated cells (1 × 10^7^) were resuspended in 400 μl culture medium supplemented with 25 mM HEPES (pH 7.4) and cooled on ice. Cold matrigel (Corning, 354277) was added and mixed with the cells at 50% volume (200 μl), then injected subcutaneously into NSG mice (JAX No. 005557) at 150 pi per injection site. Visible tumors were removed 6–18 weeks post injection, fixed in 10% Neutral Buffered Formalin, and then embedded in paraffin for staining with hematoxylin and eosin.

## Supplementary Material

1

## Figures and Tables

**Table 1:** Characterization and validation.

Classification	Test	Result	Data
Morphology	Photography	Normal	Fig. 1 Panel A
Phenotype	Immunocytochemistry	SOX2, OCT4, NANOG, SSEA-4, TRA-1-60	Fig. 1 Panel A
	Flow cytometry	TRA-1-60 (99.98%); NANOG (96.08%); SSEA-4 (99.98%)	Fig. 1 Panel B
Genotype	Karyotype (G-banding) and resolution	46XX	Fig. 1 Panel C
		Resolution: 350–400	
Identity	Microsatellite PCR (mPCR) OR	Not performed	N/A
	STR analysis	18 sites tested, all sites matched	Available with the authors
Mutation analysis (if applicable)	Sequencing	Homozygous mutation of NAGLU	Fig. 1 Panel D
	Southern Blot OR WGS	N/A	N/A
Microbiology and virology	Mycoplasma	Mycoplasma testing by luminescence. Negative Teratoma with three germlayers formation. Ectoderm (neural tube); Mesoderm (cartilage); Endoderm (gut)	Supplementary Fig. S1
Differentiation potential	Teratoma formation		Fig. 1 Panel F
Donor screening (optional)	HIV 1 + 2 Hepatitis B, Hepatitis C	N/A	N/A
Genotype additional info (optional)	Blood group genotyping	N/A	N/A
	HLA tissue typing	N/A	N/A

**Table 2:** Reagents details

Antibodies used for immunocytochemistry/flow-cytometry			
	Antibody	Dilution	Company Cat # and RRID
Pluripotency Markers	Mouse anti-SOX2	1:50	R & D systems, Cat# MAB2018, RRID: AB_358009
Pluripotency Markers	Rabbit anti-NANOG	1:400	Cell Signaling, Cat# 4903, RRID: AB_10559205
Pluripotency Markers	Rabbit anti-OCT4	1:400	Thermo Fisher, Cat# A13998, RRID: AB_2534182
Pluripotency Markers	Mouse anti-SSEA4	1:1000	Cell Signaling, Cat# 4755, RRID: AB_1264259
Pluripotency Markers	Mouse anti-TRA-1-60- Alexa Fluor 488	1:10	BD Biosciences, Cat# 560173, RRID: AB_1645379
Secondary Antibodies	Donkey anti-Mouse IgG (Alexa Fluor 488)	1:400	Thermo Fischer, Cat# A21202, RRID: AB_141607
Secondary Antibodies	Donkey anti-Rabbit IgG (Alexa Fluor 594)	1:400	Thermo Fischer, Cat# A21207, RRID: AB_141637
Flow Cytometry Antibodies	Anti-Tra-l-60-DyLight 488	1:50	Thermo Fischer, Cat# MA1-023-D488X, RRID: AB_2536700
Flow Cytometry Antibodies	Anti-Nanog-Alexa Fluor 488	1:50	Millipore, Cat# FCABS352A4, RRID: AB_10807973
Flow Cytometry Antibodies	anti-SSEA-4-Alexa Fluor 488	1:50	Thermo Fischer, Cat# 53-8843-41, RRID: AB_10597752
Flow Cytometry Antibodies	Mouse-IgM-DyLight 488	1:50	Thermo Fischer, Cat# MA1-194-D488, RRID: AB_2536969
Flow Cytometry Antibodies	Rabbit IgG-Alexa Fluor 488	1:50	Cell Signaling Technology, Cat# 4340S, RRID: AB_10694568
Flow Cytometry Antibodies	Mouse IgG3-FITC	1:50	Thermo Fischer, Cat# 11-4742-42, RRID: AB_2043894
Primers			
	Target	Forward/Reverse primer (5′-3′)	

Sev specific primers (RT-PCR)	Sev/181 bp	GGA TCA CTA GGT GAT ATC GAG C/ ACC AGA CAA GAG TTT AAG AGA TAT GTA TC	
Sev specific primers (RT-PCR)	KOS/528 bp	ATG CAC CGC TAC GAC GTG AGC GC/ ACC TTG ACA ATC CTG ATG TGG	
Sev specific primers (RT-PCR)	Klf4/410 bp	TTC CTG CAT GCC AGA GGA GCC C/ AAT GTA TCG AAG GTG CTC AA	
Sev specific primers (RT-PCR)	C-Myc/523 bp	TAA CTG ACT AGC AGG CTT GTC G/ TCC ACA TAC AGT CCT GGA TGA TGA TG	
House-Keeping gene (RT-PCR)	GAPDH/197 bp	GGA GCG AGA TCC CTC CAA AAT/ GGC TGT TGT CAT ACT TCT CAT GG	
Targeted mutation analysis (PCR)	NAGLU (c.457 G > A)/290 bp	GGG ATG GGG GAT TTG TTC/ GGC GGG TGA AAA ACA CCT AC	
