# Supplementary figures and images for: An induced pluripotent stem cell line (TRNDi006-A) from a MPS IIIB patient carrying homozygous mutation of p.Glu153Lys in the NAGLU gene
Source: Stem Cell Res. Author manuscript; Available in PMC 2019 Jun 11. (PMC6559735; doi:10.1016/j.scr.2019.101427)

## Slide 1
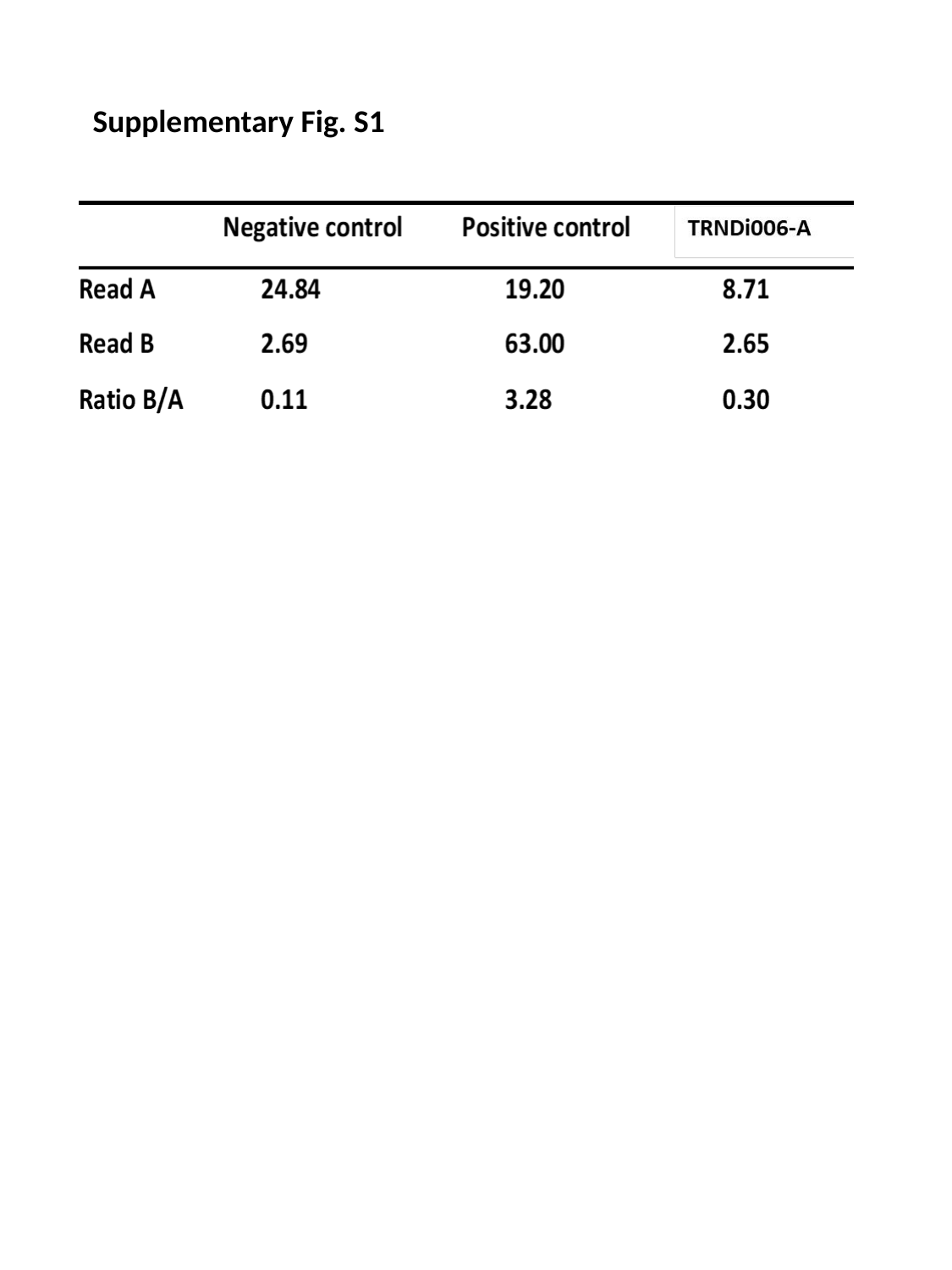

Supplementary Fig. S1

Supplement: 1 [file NIHMS1530901-supplement-1.pptx]
